# Speech Recognition and Listening Effort in Cochlear Implant Recipients and Normal-Hearing Listeners

**DOI:** 10.3389/fnins.2021.725412

**Published:** 2022-02-10

**Authors:** Khaled H. A. Abdel-Latif, Hartmut Meister

**Affiliations:** Jean-Uhrmacher-Institute for Clinical ENT-Research, University of Cologne, Cologne, Germany

**Keywords:** listening effort, speech recognition, effort scaling, dual-task, cognition, working memory

## Abstract

The outcome of cochlear implantation is typically assessed by speech recognition tests in quiet and in noise. Many cochlear implant recipients reveal satisfactory speech recognition especially in quiet situations. However, since cochlear implants provide only limited spectro-temporal cues the effort associated with understanding speech might be increased. In this respect, measures of listening effort could give important extra information regarding the outcome of cochlear implantation. In order to shed light on this topic and to gain knowledge for clinical applications we compared speech recognition and listening effort in cochlear implants (CI) recipients and age-matched normal-hearing listeners while considering potential influential factors, such as cognitive abilities. Importantly, we estimated speech recognition functions for both listener groups and compared listening effort at similar performance level. Therefore, a subjective listening effort test (adaptive scaling, “ACALES”) as well as an objective test (dual-task paradigm) were applied and compared. Regarding speech recognition CI users needed about 4 dB better signal-to-noise ratio to reach the same performance level of 50% as NH listeners and even 5 dB better SNR to reach 80% speech recognition revealing shallower psychometric functions in the CI listeners. However, when targeting a fixed speech intelligibility of 50 and 80%, respectively, CI users and normal hearing listeners did not differ significantly in terms of listening effort. This applied for both the subjective and the objective estimation. Outcome for subjective and objective listening effort was not correlated with each other nor with age or cognitive abilities of the listeners. This study did not give evidence that CI users and NH listeners differ in terms of listening effort – at least when the same performance level is considered. In contrast, both listener groups showed large inter-individual differences in effort determined with the subjective scaling and the objective dual-task. Potential clinical implications of how to assess listening effort as an outcome measure for hearing rehabilitation are discussed.

## Introduction

Cochlear implants (CI) have been established as the treatment of severe to profound hearing loss in both children and adults with hearing impairment. CIs aim at restoring hearing by means of electrical stimulation of the auditory nerve. In comparison to healthy hearing, sounds transmitted *via* CIs are largely limited especially in terms of spectro-temporal cues. Despite these limitations CIs allow open speech understanding in many patients at least in favorable surroundings (Clark, 2015).

Clinically, the functional outcome of cochlear implantation is determined by a number of measurements. In this respect, speech audiometry plays an outstanding role since it directly addresses verbal communication. Speech audiometry is typically assessed both in quiet and against background noise considering different speech materials such as phonemes, single words or sentences giving comprehensive information on speech recognition abilities (Boisvert et al., 2020).

While numerous outcome measures are established, the challenges listeners face in everyday communication are not fully addressed by common audiometric tests, since speech understanding in detrimental acoustic situations (e.g., in presence of people talking nearby, environmental sounds, or reverberation) relies not only on peripheral hearing. Amongst others, different cognitive abilities might play a role. In two meta-analyses, the role of working memory capacity (WMC) has been highlighted for listeners with healthy hearing or mild to moderate hearing loss (Akeroyd, 2008; Dryden et al., 2017). Additionally, processing speed and subdomains of executive mechanisms such as inhibitory control may play a role (Dryden et al., 2017). Less is known about the influence of cognitive factors on CI-mediated speech recognition. However, recent work has shown associations of speech recognition in CI users and in NH listeners presented with spectrally degraded (i.e., noise-vocoded) speech with WMC (Kaandorp et al., 2017), non-verbal reasoning (Mattingly et al., 2018; Moberly et al., 2018), inhibition control (Zhan et al., 2020) and processing speed as well as executive functions (Rosemann et al., 2017; Völter et al., 2021).

The role of cognition for understanding speech in adverse situations is advocated by the Ease of Language Understanding (ELU)-model (Rönnberg et al., 2013). This model postulates that understanding speech is an implicit, automated and seemingly effortless process as long as the input signal is clear. Any distortions (noise, signal processing, hearing loss) are detrimental to this process consequently activating an explicit processing putting strain on cognitive resources (i.e., working memory). Due to the generally limited capacity (Kahneman, 1973) this constitutes a cognitive load that makes performing a specific task effortful. The ELU model posits that the degree of explicit processing needed for speech understanding is positively related to effort (Rönnberg et al., 2019). Hence, it may be assumed that adverse conditions yield higher listening effort (LE) than favorable conditions despite a listener may exhibit reasonable speech recognition in both situations.

Though there is no uniform definition (McGarrigle et al., 2014) the concept of LE is increasingly common in hearing research. A number of publications define this term in the sense of the attention and cognitive resources required to understand speech (Hick and Tharpe, 2002; Fraser et al., 2010; Picou et al., 2011). The FUEL-model (“Framework for Understanding Effortful Listening,” Pichora-Fuller et al., 2016) sets a somewhat broader focus and defines listening effort as “the deliberate allocation of mental resources to overcome obstacles in goal pursuit when carrying out a task that involves listening.” Moreover, it proposes that LE depends on factors such as input-related demands (noise, signal processing, hearing loss), cognitive factors, and motivation, making it a complex multifactorial construct. According to this concept, two individuals can exhibit similar speech recognition but may differ tremendously in the effort accomplished to achieve this performance. Amongst others this might be due to differences in their cognitive abilities, as described above. For instance, Desjardins and Doherty (2013) showed that listening effort was significantly negatively correlated with working memory capacity (WMC) and processing speed. Similarly, Stenbäck et al. (2021) found a negative relation between subjectively assessed listening effort and WMC, in line with the view that larger cognitive capacity is associated with less effort. However, it should be noted that such an association was not found in all studies (cf. Rönnberg et al., 2014).

Due to the relevance of effort to daily-life communication (cf. Nachtegaal et al., 2009) and the fact that it may be related to individual factors not necessarily captured by audiometry it is reasonable to assume that determining LE could give important extra information to clinical diagnostics. In recent years there has been much research devoted to assess LE but no “gold standard” or consensus of clinical measurement has been established. Basically, subjective and objective measurements can be applied. Besides questionnaires (cf. Hughes et al., 2019) subjective measurements include rating scales (Rennies et al., 2014; Krueger et al., 2017). Mostly Likert-scales with verbal categorization ranging from “no effort” to “extreme effort” are used. Rating is typically quantified by presenting speech in the presence of a background masker with different signal-to-noise ratios (SNRs). The SNR may be adjusted adaptively in order to cover a wide range of subjectively perceived effort (“ACALES,” Krueger et al., 2017).

Objective measurements include physiological tests and behavioral performance measures. The former consider methods such as electroencephalography, pupillometry, assessment of heart rate variability, or skin conductance (e.g., Bernarding et al., 2013; Holube et al., 2016; Mackersie and Calderon-Moultrie, 2016; Winn et al., 2018) and reflect the mental load associated with listening in adverse conditions. Behavioral measures of LE are based on the fact that cognitive capacity is limited (Kahneman, 1973) and that understanding speech in detrimental situations results in fewer resources available for other tasks, in line with both the ELU- and the FUEL-model. From this rationale, listening effort can be objectively measured *via* the dual-task paradigm (Gagné et al., 2017). In this paradigm, listeners perform a primary speech recognition task simultaneously with a secondary task. In comparison to performing the tasks alone (i.e., single-task) it is assumed that the depletion of resources due to demanding listening shows up in a decline in the secondary task when keeping speech recognition stable. While the primary task typically involves presenting words or sentences in noise, a large number of secondary tasks have been proposed, both within the same modality as the primary task (i.e., auditory) as well as a different modality (i.e., tactile, visual). Moreover, secondary tasks differ largely in terms of their complexity, a factor that might affect the sensitivity of the measurements (Picou and Ricketts, 2014). Frequently, reaction times are captured for the secondary task assuming that the depletion of cognitive capacity associated with effortful listening slows down processing speed. Using these different methods it has been well established that adverse acoustic conditions, typically reflected by decreased signal to noise ratio (SNR), increase both subjectively and objectively assessed listening effort.

In the framework of clinical studies such measures of LE have also been used to assess specific signal processing strategies in cochlear implants (e.g., Stronks et al., 2020) or to compare the effort of CI recipients and NH listeners. For instance, Perreau et al. (2017) applied subjective ratings and a dual-task paradigm while modifying the SNR of the speech presented. Compared to the CI users they found a larger reduction of LE in the NH listeners when the SNR was improved suggesting that effort is different in these two groups. A meta-analysis by Ohlenforst et al. (2017) revealed that hearing-impaired persons show larger LE than normal-hearing subjects, but clear evidence was only given for electroencephalographic measures. However, Alhanbali et al. (2017) applied a subjective effort assessment scale based on six questions and also showed that hearing-impaired subjects, including groups of hearing aid and CI users, revealed significantly higher perceived effort than a control group of normal-hearing listeners. Similarly, Hughes et al. (2018) stated that hearing impaired individuals may need to invest more effort to participate successfully in everyday listening situations despite provision of hearing aids (HAs) and cochlear implants (CIs). Thus, at least during daily verbal communication hearing impaired listeners may show additional demands, even when provided with appropriate rehabilitative technologies. In terms of CIs the rationale is that the limitations in spectro-temporal processing yield extra demands that cannot readily be compensated for. Limited transmission of acoustic details in combination with adverse environments calls for cognitive compensation of speech perception constraints (Başkent et al., 2016). In line with this, pupillometry data by Winn et al. (2015) showed an impact of auditory spectral resolution beyond speech recognition when normal-hearing listeners were subjected to noise-vocoded speech aiming at simulating the spectro-temporal limits of cochlear implants. In contrast, it has also been shown in adolescent CI and NH listeners that both groups show similar effort once performance has been balanced (Hughes and Galvin, 2013). Thus, it remains unclear if and under what circumstances hearing impairment and CI-mediated listening yield increased effort.

In the present study, we compared listening effort in experienced CI recipients and age-matched NH listeners while considering potential influential factors, such as cognitive abilities. Based on the outcome of this comparison we discuss implications for the use as a clinical outcome measure. To this end two measurements of listening effort previously applied in clinical studies, a subjective scaling procedure as well as an objective test (dual-task paradigm), were applied and compared. Importantly, we estimated speech recognition functions for both listener groups and contrasted listening effort at similar performance levels. We hypothesized that listening effort is higher for CI users than NH listeners due to the degraded signal conveyed by the CI and that individual cognitive abilities of the participants mediate listening effort.

## Materials and Methods

### Participants

Two groups (*n* = 14 each) of cochlear implant users with at least 2 years of CI experience and age-matched NH listeners were recruited for participation in this study. The CI recipients used different devices and all except three were fitted bilaterally. Detailed information is given in Table 1. The NH listeners had pure tone thresholds ≤ 25 dB HL across all frequencies of 125 to 4,000 Hz and were chosen to match the age of the CI users as closely as possible. The NH group involved 11 female and 3 male listeners. The maximum age difference between each CI-NH pair was 3 years. Thus, both groups did not differ regarding their age (61.9 ± 12.4 years for CI and 62.4 ± 12.6 years for NH). All participants were native German speakers and had normal or corrected-to-normal vision. Prior to the experiment they were given detailed information about the study and informed consent was obtained. Participants were reimbursed with € 10,-/h. The study protocol was approved by the local ethics committee.

**TABLE 1:** Characteristics of the cochlear implant recipients.

ID	Gender	Age (years)	Fitting	Hearing loss right ear since (years)	Hearing loss left ear since (years)	Experience right CI (years)	Experience left CI (years)	Word recognition score (%)	Right CI type	Left CI type
1	m	47	bilateral	22	22	4	4	90	Cochlear^®^ N6	Cochlear^®^ N6
2	m	67	bilateral	childhood	childhood	14	19	45	Advanced Bionics, Auria (SAS)	Advanced Bionics, Auria (HiRes-P)
3	f	74	bilateral	40	35	15	8	85	Advanced Bionics, Naída CI Q90	Advanced Bionics, Naída CI Q90
4	m	83	unilateral	childhood	childhood	–	16	70	–	MED-EL, Sonnet
5	f	68	unilateral	na	na	10	–	55	MED-EL, Opus2	–
6	f	59	bilateral	41	41	6	4	80	MED-EL, Opus2	MED-EL, Opus2
7	m	71	bilateral	childhood	childhood	2	11	90	MED-EL, Sonnet	MED-EL, Opus2
8	f	57	bilateral	32	32	7	3	75	Cochlear^®^, CP810	Cochlear^®^, CP810
9	m	60	unilateral	18	18	16	–	60	Cochlear^®^, CP910	–
10	f	61	bilateral	41	41	19	8	85	Advanced Bionics, Harmony	Advanced Bionics, Harmony
11	f	52	bilateral	47	47	5	6	90	MED-EL, Opus2	MED-EL, Opus2
12	f	78	bilateral	na	na	11	4	55	MED-EL, Sonnet	MED-EL, Sonnet
13	m	39	bilateral	18	18	3	3	90	Cochlear^®^, CP910	Cochlear^®^, CP910
14	f	51	bilateral	26	26	5	5	90	MED-EL, Opus2	MED-EL, Synchrony

### Cognitive Tests

As described in the introduction several cognitive functions are potentially related to recognizing speech in adverse conditions as well as the associated listening effort. From the variety of these functions we selected three that are suited for clinical assessment based on appropriate neuropsychological tests.

Working memory capacity (WMC) was assessed by the German version of the Reading Span Test (RST; Carroll et al., 2015). This test presents sentences in blocks of 2 to 6 stimuli on a computer screen. The task is to read each sentence aloud and to judge immediately after presentation whether the sentence is meaningful or not. At the end of each block, the participant is asked to recall the first or last word of the sentences. The percentage of correctly recalled words across all trials is determined and taken as an indicator of WMC.

Furthermore, processing speed and executive functions were assessed by the Trail Making Test (TMT; Reitan, 1958). The TMT consists of two subsets: In TMT-A the participants are asked to connect digits shown on a sheet of paper in ascending numerical order. In TMT-B the participants are required to alternate between digits and letters in ascending order. In both tests the time to complete the task is assessed. TMT-A and TMT-B are thought to give an indication of different cognitive abilities (Sanchez-Cubillo et al., 2009). Specifically, TMT-A is associated with processing speed and TMT-B is assumed to reflect executive control and cognitive flexibility.

### Speech Recognition in Noise

The Oldenburg sentence test (OLSA, Wagener et al., 1999) was used for assessing speech recognition in noise. This test is frequently applied in clinical routine in Germany. The OLSA is a matrix test presenting sentences composed of five words (name – verb – numeral – adjective – object) and ten possible alternatives for each word position. Sentences are syntactically correct but semantically unpredictable thus allowing repeated testing. The male voice of the OLSA was used. The masker was a test-specific stationary noise (“olnoise”) generated by multiple random superpositions of the sentences of the OLSA corpus. These stimuli were used for examining speech recognition as well as for the subjective and objective assessment of listening effort.

An important aspect of the study was to estimate the speech recognition function of the listeners. To this end the 50% speech recognition threshold (SRT50) as well as the slope of the recognition function were assessed concurrently following the procedure suggested by Brand and Kollmeier (2002). This procedure adaptively tracks correct response probabilities of 19 and 81% in an interleaved fashion during one test list of 30 trials. Initial step-width for varying the SNR is 1.5 dB and reduced after each reversal yielding a final step-width of 0.25 dB to stabilize presentation levels near the targets. The SNRs presented after five reversals of the adaptive procedures were averaged to determine the two targets. Based on the estimates of 19 and 81% intelligibility the SRT50 and the slope are determined. The noise was fixed at 65 dB SPL and the speech level was varied depending on the subject’s responses, who were asked to repeat back as many words as possible. The stimuli were routed from a PC to an audiometer (Siemens Unity) and sent to a free-field loudspeaker (Events Electronics, Australia) placed at a distance of 1.2 m from the listener’s head located at 0°. In order to test reliability and to improve accuracy of the psychometric function this measurement was performed twice using test lists of 30 sentences each. Based on the individual threshold and the slope derived from the measurements a logistic function


(1) $$y=\frac{100}{1+e^{-\frac{\left(x-S\,R\,T\,50\right)}{s}}}$$

was fitted, with SRT50 as the SNR associated with 50% intelligibility, s as the slope at 50% intelligibility, x as the level in dB SNR, and y as the percentage of words correctly understood.

This function was used to estimate the SNR associated with 80% intelligibility that was applied for assessing objective listening effort in the dual-task paradigm.

### Objective Listening Effort

Listening effort was measured with a dual-task paradigm, consisting of a listening task (primary task) and a visual reaction time task (secondary task). This behavioral paradigm determines performance and thus assesses effort objectively. The primary task was to recognize speech at a performance level of 80%. Choosing this level represented a situation where performance was relatively high but still demanding and followed the recommendation to avoid unfavorable SNRs with dual-task paradigms in order to prevent cognitive overload (Wu et al., 2016). Since it was difficult to target exactly 80% for each listener a range of ±8% was allowed. This range of maximum 16% was not expected to have a significant influence on listening effort, in line with the psychometric functions of dual-task paradigms given in Wu et al. (2016). If this criterion was not met the SNR was readjusted and the measurement was repeated until the desired range was reached. This was necessary in seven cases.

The secondary task was a visual reaction time task. We chose a simple task in order to maximize the possibility that the primary task was unaffected. A white fixation cross (visual angle = 5.2°) was shown on a black background *via* a computer screen (ELO TouchSystems) placed about 65 cm in front of the subject. The cross briefly disappeared at arbitrary points in time during the presentation of half of the sentences of a test list at random intervals. The task of the participants was to react as fast as possible by pressing the left mouse button.

The dual-task paradigm was administered using a custom made computer program, implemented using the *Presentation* software (Neurobehavioral Systems Inc., Berkeley, CA, United States). Sentences of the OLSA masked by olnoise were sent *via* an external sound-card (Hammerfall DSP Multiface II) to the loudspeaker as described with the speech recognition procedure.

The primary and secondary tasks were measured separately *via* single-task, as well as in a combined fashion *via* dual-task. The single-task measurements served as baselines. Here, the participants were asked to concentrate on the task at hand (speech recognition or visual reaction) and to ignore the other task (visual reaction or speech recognition). In the dual-task instructions were given to the participants to optimize performance in the primary task (speech recognition) but also to perform the secondary task as accurately and fast as possible (cf. Gagné et al., 2017). In each condition test lists of 40 sentences were presented. Because in the secondary task only half of the stimuli were randomly associated with the fixation cross disappearing, twenty reaction time scores were recorded across a test list. Since reaction times typically show a non-normal distribution a median score was calculated across a test list for each participant.

In order to derive a measure of listening effort, proportional dual-task costs (pDTC%) indicating the load on the secondary task (Fraser et al., 2010) was calculated by the formula


(2) pDTC%=100*(Secondary(dualtask)-Secondary(singletask))/Secondary(singletask)

Likewise, proportional dual-task costs can be calculated for the primary task. However, as intended and shown below, the primary task was not critically affected by combining both tasks.

### Subjective Listening Effort

Listening effort was measured subjectively with the “Adaptive Categorical Listening Effort Scaling” (ACALES, Krueger et al., 2017). Similar to the speech recognition test this method presents sentences of the OLSA masked by olnoise at various SNRs. Again, stimuli were sent *via* an external sound-card (Hammerfall DSP Mulitface II) to the loudspeaker as described above. With each SNR two sentences were presented allowing a reasonable amount of time to listen to the stimuli. After each presentation the listeners were asked to answer the question “How much effort does it require for you to follow the speaker?” (German: “Wie anstrengend ist es für Sie, dem Sprecher zu folgen?”). LE is assessed on a categorical scale showing the labels “no effort,” “very little effort,” “little effort,” “moderate effort,” “considerable effort,” “very much effort,” “extreme effort,” displayed on a touch screen (ELO TouchSystems). These labels corresponded to 1, 3, 5, 7, 9, 11, and 13 effort scale categorical units (ESCU), respectively. There were six unlabelled intermediate steps and an additional category (“only noise”) that allowed for a response when no speech was perceived. The ESCU-values were not shown to the subjects.

The adaptive procedure consists of three phases (details in Krueger et al., 2017). In the first phase the boundaries for “no effort” and “extreme effort” are searched by varying the SNR by a step-width of 3 dB. These boundaries are used for the second phase that presents five intermediate SNRs to estimate the five categories “very little effort,” “little effort,” “moderate effort,” “considerable effort,” and “very much effort.” By linear interpolation of these data the SNRs for “no” and “extreme effort” are re-estimated and SNRs for the five intermediate categories are re-calculated and presented to the listeners in a third phase. Based on these presentations LE estimates were determined by linear regression for each listener.

### Procedures

After giving informed consent the participants first completed the cognitive tests beginning with the TMT and followed by the RST. Speech recognition testing and listening effort experiments were run in a sound treated booth (l:4 × w:3 × h:2 m). Speech recognition in noise was preceded with a training phase presenting two tests lists of 20 sentences each in quiet in order to familiarize the participants with the OLSA-material. After that, subjective listening effort was assessed. Prior to the measurement a short training by presenting 20 stimuli at different SNRs was performed in order to familiarize the participants with the method and the rating scale. Finally, the dual-task paradigm was performed in order to assess listening effort objectively. Again, prior to conducting the actual experiment a training phase familiarized the subjects with the tasks and the stimuli provided. Testing was accomplished in a single visit lasting approximately 3 h, including several individual breaks.

### Statistical Analyses

Kolmogorov-Smirnov-Tests and visual inspection of Q-Q-plots revealed that the data were mostly normally distributed. In that case, repeated measures analyses of variance (rmANOVA) were performed. If the assumption of sphericity was violated, Greenhouse-Geisser corrections were used. The association of listening effort outcome and cognitive tests was assessed by correlation analysis. In the case of non-normally distributed data non-parametric tests were used as documented in the results section. IBM SPSS v. 25 was used for all calculations.

## Results

### Speech Recognition in Noise

Individual speech recognition functions were estimated based on the procedure described above. Test and retest were highly correlated (Pearson’s coefficients *r*_*p*_ = 0.95 for SRT50, *r*_*p*_ = 0.83 for slope, both *p* < 0.001) and thus outcome was averaged across the two measurements. Hence, estimates of the functions were based on 60 sentences in total.

**FIGURE 1:**
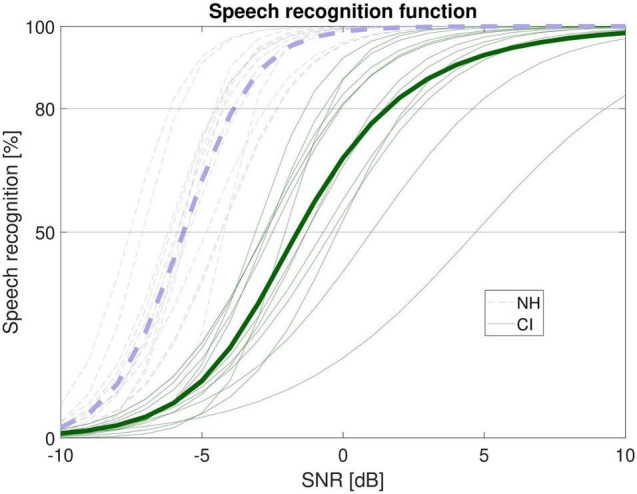
Estimated speech recognition functions for the CI recipients (green) and the NH listeners (violet). Thin lines show individual functions, bold lines show the group mean. SNR = signal-to-noise ratio.

Figure 1 shows the individual functions of both listener groups. As expected, speech recognition was clearly better for the NH than the CI listeners. A rmANOVA on SNR with target speech recognition (50%, 80%) as within-subjects variable and listener group (CI, NH) as between-subjects variable revealed a significant main effect of target speech recognition (*F*_1,26_ = 338.96, *p* < 0.001, η*_p_*^2^ = 0.93), a significant main effect of group (*F*_1,26_ = 49.52, *p* < 0.001, η_*p*_^2^ = 0.66) and a speech recognition by group interaction (*F*_1,26_ = 23.65, *p* < 0.001, η_*p*_^2^ = 0.48). The mean SNR associated with 50% recognition was −5.6 ± 0.9 dB SNR in the NH listeners and −1.2 ± 2.0 dB SNR in the CI listeners. The estimation of 80% speech recognition revealed a SNR of −4.1 ± 1.1 dB SNR in the NH listeners and +1.4 ± 2.7 dB SNR in the CI users. Follow-up of the significant interaction revealed that the difference in SNR between 50 and 80% target speech recognition was significantly larger in the CI listeners than in the NH listeners (*t*_1,26_ = 4.86, *p* < 0.001). This shows that the slope of the function was typically steeper in NH than CI listeners.

Further analyses revealed that SRT50 and slope were significantly correlated in the CI recipients (*r*_*p*_ = −0.71, *p* = 0.005) but not in the NH listeners (*r*_*p*_ = −0.26, *p* = 0.372) which might be attributed to the relatively low variability in speech recognition in the latter group. However, for the CI users it could be approximated that the slope changed by about 1% per dB/SRT, which might be helpful for estimating speech recognition at different SNRs.

### Subjective Listening Effort – ACALES

**FIGURE 2:**
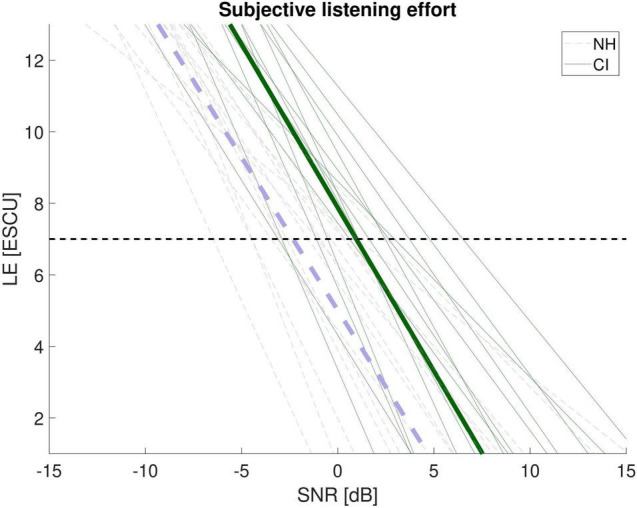
Listening effort (LE) assessed by ACALES as a function of the signal-to-noise ratio (SNR) in CI recipients (green) and NH listeners (violet). Bold lines represent the mean. ESCU = Effort Scaling Category Units. The dashed horizontal line shows the value of 7 ESCU (“moderate effort”).

For each participant listening effort outcome was fitted by a simple linear regression function which is suitable when using a stationary test-specific masker (i.e., olnoise, see Krueger et al., 2017). Figure 2 shows the results for both listener groups in dependence of the SNR applied. While the slope of the functions is similar for NH and CI listeners (*t*_1,26_ = 0.11, *p* = 0.91) the value for LE7 as the proxy for moderate effort (i.e., 7 ESCU) is significantly different (*t*_1,26_ = 3.2, *p* = 0.004). As shown in the figure both group-mean functions are shifted by about 3 dB SNR given the same ESCU-value or about 3 ESCU given the same SNR.

**FIGURE 3:**
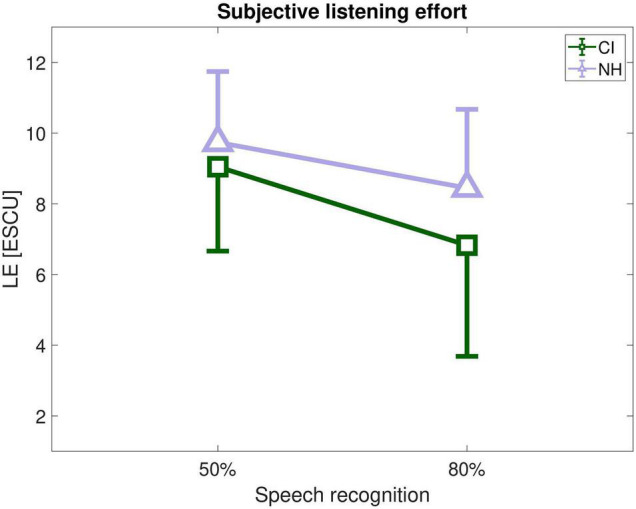
Listening effort (LE) in CI recipients (green) and NH listeners (violet) at SNRs associated with 50 and 80% speech recognition. ESCU = Effort Scaling Categorical Units.

By using the estimated speech recognition functions (see Figure 1), individual LE-scores for 50% and 80% speech recognition, denoted as LE50 and LE80 were determined (see Figure 3). Mean listening effort was about 9–10 ESCU (“considerable” to “very much effort”) for 50% speech recognition and around 7–9 ESCU (“moderate” to “considerable effort”) for 80 % recognition. A rmANOVA with speech recognition (50%, 80%) as within-subjects variable and listener group (CI, NH) as between-subjects variable revealed a significant main effect of speech recognition (*F*_1,26_ = 130.35, *p* < 0.001, η_*p*_^2^ = 0.83) and a significant speech recognition by group interaction (*F*_1,26_ = 11.81, *p* = 0.002, η_*p*_^2^ = 0.31). The interaction mirrored the impression of Figure 3 that CI and NH listeners rated LE relatively similar at 50% but NH perceived somewhat higher LE at 80%. However, *post hoc* tests rendered this group difference insignificant (*t*_1,26_ = −1.94, *p* = 0.064).

### Objective Listening Effort

**FIGURE 4:**
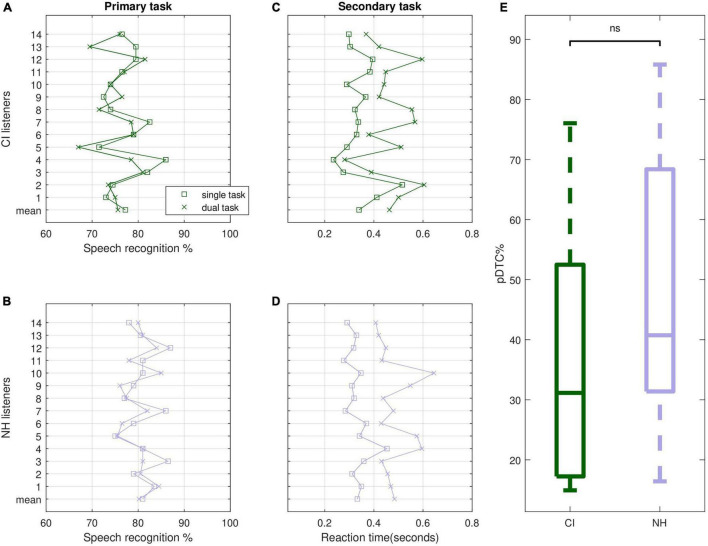
Outcome of the dual-task paradigm. **(A,B)** Primary task (speech recognition) in CI and NH listeners, **(C,D)** secondary task (reaction time) in CI and NH listeners, **(E)** proportional dual-tasks costs for the secondary task. CI, cochlear implant recipients; NH, normal-hearing listeners. Squares = single-task, crosses = dual-task.

The primary task of the dual-task paradigm showed that the goal to target a speech recognition of about 80% was met in both listener groups (Figures 4A,B). Apart from single cases (CI05, CI13) this held for both, performance in the single-task and the dual-task condition. A rmANOVA with task (single, dual) as within-subjects variable and listener group (CI, NH) as between-subjects variable revealed a significant main effect of task (*F*_1,26_ = 4.85, *p* = 0.037, η_*p*_^2^ = 0.16) and a significant main effect of group (*F*_1,26_ = 9.56, *p* = 0.005, η_*p*_^2^ = 0.27). Speech recognition was higher in the single-task than in the dual-task (79.1 ± 4.2% vs. 77.7 ± 4.1%) and in the NH compared to the CI listeners (80.5 ± 3.1% vs. 76.7 ± 4.2%). Since our aim was to capture LE by dual-task costs in the secondary task, as outlined above, a performance difference in the primary task could be critical. However, despite statistical significance this difference did not influence outcome, as proportional dual-task costs for the primary task amounted to only about 2%, when calculated in analogy to formula (2). Furthermore, based on the psychometric functions of dual-task paradigms given in Wu et al. (2016), it is assumed that the small performance difference between CI and NH listeners of about 4% in the primary task did not affect costs in the secondary task.

Reaction times in the secondary task were highly variable and appear to show a clear delay in all cases, when assessed in the dual-task (see Figures 4C,D). Subjecting the data to a rmANOVA with task (single, dual) as within-subjects variable and listener group (CI, NH) as between-subjects variable revealed a significant main effect of task (*F*_1,26_ = 110.30, *p* < 0.001, η_*p*_^2^ = 0.81). Corresponding proportional dual-task costs are shown in Figure 4E. Comparing the costs between both listener groups revealed no significant difference (*U*-Test, *z* = 1.15, *p* = 0.27).

### Listening Effort and Cognitive Functions

The listeners of both groups were assessed in terms of their processing speed, cognitive flexibility, and working memory capacity using the Trail making Test (Version A and B) and the German version of the Reading span test (Carroll et al., 2015). Outcome is given in Table 2.

**TABLE 2:** Outcome of the neuropsychological tests regarding processing speed (TMT-A), executive control (TMT-B), and working memory capacity (RST).

		Minimum	Maximum	Median	Mean	Std.-Dev.
CI-recipients (*n* = 14)	TMT-A [s]	16	76	32.8	39.9	19.1
	TMT-B [s]	36	393	72.0	105.7	105.3
	RST [%]	11.1	66.6	42.5	39.6	14.5
NH-listeners (*n* = 14)	TMT-A [s]	21	77	35.9	40.7	18.6
	TMT-B [s]	21	128	68.6	76.2	31.6
	RST [%]	24.1	61.1	41.6	40.8	8.8

The CI recipients revealed two outliers for the outcome of TMT-B. Groups were compared using *U*-tests that did not show any significant difference for the tests applied (all *p* > 0.45).

Table 3 shows the Spearman’s correlation coefficients of the neuropsychological test outcome and the listening effort measures across both groups. LE80 was taken as the proxy for subjective listening effort and pDTC% as the proxy for objective listening effort, both reflecting the demands associated with 80% speech recognition. Age of the listeners was also considered as it is assumed to be associated with cognition. Indeed, TMT-A, TMT-B, and RST showed a significant correlation with age. As expected, older listeners were slower in both Trail making tests A and B and showed worse recall in the WMC test. Furthermore, the three cognitive metrics were significantly correlated demonstrating that they do not represent completely unrelated domains. This also held when the two outliers (TMT-B) were removed.

**TABLE 3:** Spearman’s rank correlations and significance levels (two-tailed) of the outcome of the listening effort measures (LE80, pDTC%) and neuropsychological tests (TMT-A, TMT-B, RST), as well as age, Asterisk depict significant correlations.

		LE80	pDTC%	TMT-A	TMT-B	RST	Age
LE80	r_*sp*_	1.000	–0.100	–0.104	–0.021	0.105	–0.332
	p		0.612	0.598	0.916	0.595	0.085
pDTC%	r_*sp*_		1.000	0.169	–0.221	–0.151	0.225
	p			0.389	0.258	0.443	0.250
TMT-A	r_*sp*_			1.000	0.805**	−0.556**	0.695**
	p				0.000	0.002	0.000
TMT-B	r_*sp*_				1.000	−0.529**	0.556**
	p					0.004	0.002
RST	r_*sp*_					1.000	−0.583**
	p						0.001
Age	r_*sp*_						1.000
	p						

However, both LE80 and pDTC% did not reveal any significant correlation with the outcome of the neuropsychological tests nor with age. Moreover, the two LE outcome measures were not significantly associated with each other suggesting that they tap into different dimensions of the listening effort construct.

## Discussion

The aim of this study was to compare measures of listening effort and speech recognition in CI recipients and age-matched normal-hearing listeners and to gain information for potential clinical applications and implications. To this end, methods that potentially may be used in clinical assessments were considered. We hypothesized that CI recipients show increased effort due to the limitations of CI-mediated sound transmission. Alternatively, it could be suspected that CI and NH listeners exhibit comparable listening effort once speech recognition performance of the participants is balanced. Furthermore, we expected that individual cognitive abilities may mediate listening effort.

### Speech Recognition in Noise

Paramount to our examination of LE was that individual speech recognition performance in noise was known. Therefore, speech recognition functions were estimated. As expected, the functions revealed better performance in the NH than the CI listeners. This manifested in both, speech recognition thresholds and slope of the functions. The latter was shallower for the CI users, that is, they did not benefit from increasing the SNR to the same amount as the NH listeners. This confirms results by MacPherson and Akeroyd (2014) who found a trend of decreasing slope with increasing hearing impairment. Moreover, Sobon et al. (2019) reported a significant negative correlation between slope and SRT in NH listeners, but only for a two-talker speech masker. In general, one single SRT (typically associated with 50% recognition) may thus not fully acknowledge speech recognition problems over a wider range of SNRs. However, the decrease in slope of about 1% per dB SRT in the CI listeners might be helpful for estimating performance at different SNRs. From a practical background this indicates that listeners with poor SRTs may gain less from any change in SNR offered by the signal processing in hearing aids or cochlear implants (cf. MacPherson and Akeroyd, 2014).

Thus, from a clinical perspective it seems advisable to determine not only the SRT but also the slope. According to Brand and Kollmeier (2002) this is basically feasible by using a test list of at least 30 sentences. These “extra costs” appear to be acceptable in the framework of clinical routine where typically at least 20 sentences (in the case of matrix sentences after training) are used. Hence, the proposed method of assessing both, SNR and slope might give valuable extra information, especially when trying to relate other measures (such as listening effort outcome) to individual speech recognition, as will be discussed in the following.

### Subjective Listening Effort

Assessing subjective listening effort, e.g., *via* ACALES, appears to be easily applicable in clinical routine. Methodological demands and time consumption are moderate. Determining listening effort including a brief orientation phase takes about 6–8 min. Clear instructions provided, the procedure appears to be a good representation of what it intends to measure. Thus, it may be assumed that it reveals high face validity. In terms of reliability, Krueger et al. (2017) reported a high intraclass correlation above 0.9 when using the olnoise masker. However, since each listener might have his or her own subjective effort construct, it is not entirely clear whether individual outcome mirrors the same underlying dimensions and whether results can be directly compared with each other. Potentially as a consequence, estimated LE showed high interindividual variability in both, CI and NH listeners.

ACALES assesses subjective LE relative to adaptive variations in SNR. This has the advantage that the entire range from “no” to “extreme effort” is covered. When relating LE to SNR there was indeed a significant difference between the listener groups. NH participants showed about 3 ESCU lower listening effort ratings for the same SNR. However, this comparison might be misleading if the association of SNR with speech recognition is unknown. In the present study this association could be estimated based on the individual psychometric functions of the participants. When similar performance was assumed, both groups did not differ significantly with respect to LE. Nevertheless, a significant speech recognition by group interaction was found reflecting that CI users exhibited lower effort at 80% performance relative to the NH listeners (see Figure 3). Despite *post hoc* tests rendered this difference insignificant (*p* = 0.064) it deserves further discussion. In general, it is not exactly clear which factors contribute to the individual estimation of listening effort. However, it is conceivable that the subjectively perceived level of the speech signal relative to the noise is taken into account. Due to the shallower speech recognition function in the CI recipients SNR improved more than in the NH listeners when targeting 80% recognition instead of 50%. This would be in line with the observation of a larger decrease in ESCU in the CI users than in the NH listeners.

### Objective Listening Effort

Assessing listening effort objectively typically assumes high methodological and technical demands, as it is the case with electroencephalography, pupillometry, electrodermal activity or heart rate variability (cf. Bernarding et al., 2013; Holube et al., 2016; Mackersie and Calderon-Moultrie, 2016; Winn et al., 2018). In terms of behavioral measurements an alternative are dual-task paradigms which consist of a primary and a secondary task. The reliability of dual-task paradigms appears to be satisfactory, as Picou and Ricketts (2014) reported a test-retest correlation of 0.79 when using a “simple” secondary task comparable to that of the present study. However, in contrast to the subjective estimation it has to be taken into account, that time consumption is about three times higher (20–25 min), since three test lists have to be administered successively.

The primary task was recognizing speech at a SNR associated with 80% performance. This level was considered in order to make the task demanding but to avoid low performance that might be detrimental to these paradigms due to cognitive overload (see Wu et al., 2016) and also to better reflect everyday listening where intelligibility is mostly high or approaches ceiling. The results presented above confirm that 80% recognition was related to substantial subjective effort. Ideally, the performance in the primary task is constant across all test conditions since the proxy for LE is expected to emerge in the secondary task. Our statistical analysis of the primary task outcome revealed significant condition- and group-effects. However, these differences were in a range of only a few percent and are assumed not to play a critical role regarding the task load. Thus, the goal of keeping the primary task relatively constant across listeners and tasks and capturing the effect of dual-task costs in the secondary task appears to be met.

Significant proportional dual-task costs reflecting listening effort could be shown in the secondary task. Costs showed large interindividual differences but both listener groups did not differ significantly which also supports the idea that LE is similar when comparable speech recognition is assumed. In this study, we applied a simple reaction-time based secondary task providing 20 RTs across one test list. This is a relatively low number potentially affecting the quality of the outcome. However, when assessing split-half reliability (i.e., trials 1–10 vs. trials 11–20) the correlation was high (*r*_*p*_ ≥ 0.8, *p* < 0.001) for both the primary and the dual-task. Moreover, calculating the average RTs across groups revealed very similar results, regardless of whether the first or second half of trials was used.

The choice of the secondary is generally critical. On the one hand it must not be too demanding in order to avoid performance shifts across tasks (“trade-off”) and on the other hand it must not be too simple because of the then missing task load. In our case, the choice of a relatively simple visual paradigm appears to be appropriate, since the primary task outcome remained largely stable and load effects clearly surfaced in the secondary task. However, a secondary task requiring more processing depth might be even more sensitive. Picou and Ricketts (2014) compared different secondary tasks, involving a simple and a complex visual reaction time paradigm as well as a semantic paradigm, requiring to understand the word presented in the primary task. Whereas the visual reaction time paradigms both reflected the effect of background noise on LE the latter showed larger effects sizes and thus might better reflect more subtle mechanisms of effort. Further, Hsu et al. (2020) modified the depth of processing in the secondary task by asking children with CIs to judge whether the word presented was an animal (lower level of semantic processing) or whether the animal was dangerous (higher level). However, both secondary tasks appeared to reflect the increased load associated with adding noise (i.e., SNR of 3 dB) relative to listening in quiet.

### Association With Cognition and Age

Three cognitive domains (processing speed, executive control and working memory capacity) potentially associated with recognizing speech and listening effort in adverse acoustic situations were considered. No significant group effects were found. This does not support the expectation that hearing impaired persons show lower cognitive abilities compared to age-matched normal-hearing listeners (e.g., Lin et al., 2013). However, as expected, the outcome of the cognitive tests was correlated with age. Nevertheless, none of the cognitive metrics nor age was significantly associated with subjectively (i.e., LE80) or objectively (pDTC%) assessed listening effort. This finding was unexpected, given the theoretical rationale that effortful listening depletes limited cognitive resources, as proposed by the ELU- and the FUEL-model.

Reports on the correlation of listening effort outcome and cognitive abilities are relatively scarce. Harvey et al. (2017) found that cognitive functions predict listening effort performance during complex tasks in NH listeners. Furthermore, Hua et al. (2014) showed that participants with better cognitive flexibility reported less perceived listening effort. In contrast, Brännström et al. (2018) reported no significant association of measures of WMC and cognitive flexibility with subjectively perceived effort. However, they found a positive correlation of listening effort and inhibitory control. This result was surprising, given that better inhibitory control was associated with higher perceived effort. In listeners provided with cochlear implants, Perreau et al. (2017) also did not find an association of WMC and LE in a dual-task paradigm, but age and LE were correlated. However, as recently pointed out by Francis and Love (2020), LE suggests a complex and possibly “unresolvable” interaction between the commitment of processing resources on the one hand and the response to their deployment on the other hand.

The proxies of subjective and objective listening effort also did not show a significant relation with each other. While some examinations report correlations for single factors (e.g., Holube et al., 2016; Picou and Ricketts, 2018) this is generally in line with a number of studies showing a lack of correspondence between objective and subjective measures of listening effort (e.g., Fraser et al., 2010; Zekveld et al., 2010; Gosselin and Gagné, 2011) and is consistent with the assumption that measures of LE are multidimensional (McGarrigle et al., 2014; Alhanbali et al., 2019). In this context, Lemke and Besser (2016) distinguish between perceived listening effort and processing load. Following this view applying the ACALES procedure addresses perceived LE whereas the dual-task rather reflects the latter. As pointed out by Lemke and Besser (2016), a listening situation might pose high processing load but must not necessarily be perceived as effortful, and vice versa.

### General Discussion

Including listening effort in the assessment of hearing disorders could add a dimension that has not yet been covered by clinical auditory measurements. It could also provide information regarding rehabilitative measures such as the use of specific signal processing or training programs. As discussed above the two measurements of LE applied in this study appear to tap into different domains of the listening effort framework. Both, estimating subjectively perceived listening effort, e.g., *via* ACALES as well as the dual-task paradigm do not require much technological or organizational resources and can be readily integrated using standard speech audiometric material. Another important clinical criterion is the time required to perform the measurement. In this respect the adaptive ACALES procedure appears to be better suited than a the dual-task paradigm, which contains three successive test lists. As a matter of fact, however, extra information can only be gained when additional time is allowed.

Independent from the method used we hypothesized that CI listeners reveal larger LE compared to NH subjects. This was indeed the case when subjective LE was related to the SNR. However, it did not hold when balancing performance across listener groups. This is in line with Hughes and Galvin (2013) who also demonstrated similar LE in adolescent CI recipients and normal-hearing subjects when similar speech recognition was considered.

In general, a close connection of LE and speech recognition performance could be demonstrated. It is tempting to review some recent studies on listening effort in cochlear implant recipients in the light of the present findings. For instance, Perreau et al. (2017) assessed LE subjectively as well as objectively in different groups of CI users and a control group of normal-hearing listeners. The objective measure of LE based on a dual-task paradigm including a reaction-time metric. The authors considered six different SNR-conditions revealing speech recognition scores from around 60% to near perfect. Across the SNR conditions they found larger reduction in LE for the NH compared to the CI listeners. However, considering the steeper psychometric function of normal-hearing listeners as described above, this finding may be explained by their larger increment in performance for a given SNR increase than for the CI recipients.

The effect of a specific sound processing algorithm (i.e., “soft voice”) on speech recognition and listening effort was examined by Stronks et al. (2020). The algorithm aims at improving speech recognition at low sound levels by removing internal noise of the device. LE was assessed objectively by pupillometry and subjectively by scaling. Whereas pupillometry did not reveal any effect of the processing algorithm, it had a positive effect on subjectively perceived effort at a speech level of 33 dB SPL (SNR = −5 dB). This was also the level where the algorithm improved speech recognition to the largest extent, giving evidence for a close connection of performance and LE. Consequently, the authors stated that performance measures themselves might be a valid predictor of listening effort. Thus, as outlined in the present study, effects on LE might be difficult to interpret if the underlying speech recognition performance is unknown.

In terms of clinical applications this also raises the question in which cases LE measurements actually provide extra information over commonly used speech audiometry. Given the typical time limitations in clinical assessments this question is crucial. In the present study it could be shown that at least over a range of 50 to 80% speech recognition a close connection between performance and LE can be found. Moreover, no differences in LE between CI and NH listeners were found once performance was accounted for. Most of the studies that assumed larger LE for listeners with hearing loss referred to everyday listening, that is, situations typically including positive SNRs and high speech intelligibility (Smeds et al., 2015). In this regard the matrix-test reveals limited ecological validity, since the SRTs determined are often in a negative SNR-range. The functions presented in Figure 1 show that all NH listeners show perfect speech recognition at positive SNRs whereas some of the CI users approach asymptote at higher signal-to-noise ratios. Thus, it is plausible that CI recipients show increased effort at these ecologically more valid SNRs. This is also confirmed when looking at the association of LE and signal-to-noise ratio depicted in Figure 2. This suggests that assessing LE might provide more information when it is not assessed at 50 or 80% speech intelligibility but rather when speech recognition is near or at ceiling. Here, LE stills shows considerable inter-individual variability though effort is lower than at intermediate speech recognition. However, sustained effort could still yield substantial fatigue (Hornsby et al., 2016). Thus, even differences in low effort may have practical consequences for everyday life. Moreover, particular signal processing schemes such as noise reduction algorithms may not affect intelligibility but could be efficient regarding the reduction of effort.

## Conclusion

There is increasing need for measures that capture effects of speech perception beyond speech audiometry. This is due to advances in rehabilitation technology and the fact that challenges in everyday communication are not fully covered by common audiometric tests. One construct that promises valuable information is the effort associated with recognizing speech. Here, we compared the results of two potentially clinically suited methods in groups of listeners with cochlear implants and normal hearing. Both measurements revealed highly variable results that were not significantly related to different cognitive abilities or age. Moreover, the outcome of the two tests was not correlated with each other suggesting that they tap into different dimensions of the effort construct. Also, we did not find any significant difference in LE between the two listener groups, once performance was equalized by adjusting individual SNRs. A limitation of the study was that the sample size of the two groups was small and thus might not have been sufficient to detect small effects. However, LE was strongly correlated with speech recognition at least when assessed subjectively. Thus, when examining LE it is highly recommended to take possible performance differences into account, e.g., by determining both, SRT and slope of the psychometric function. Due to the strong association of effort and speech recognition it is suggested that LE-assessment is more instructive when performance is near or at ceiling. Here, the large inter-individual variability in listening effort could give information beyond speech audiometry and would also consider the range of more ecological signal-to-noise ratios.

## Data Availability Statement

The raw data supporting the conclusions of this article will be made available by the authors, without undue reservation.

## Ethics Statement

The studies involving human participants were reviewed and approved by the Ethics Committee of the Medical Faculty, University of Cologne. The patients/participants provided their written informed consent to participate in this study.

## Author Contributions

Both authors have made a substantial, direct, and intellectual contribution to the work and approved it for publication.

## Conflict of Interest

The authors declare that the research was conducted in the absence of any commercial or financial relationships that could be construed as a potential conflict of interest.

## Publisher’s Note

All claims expressed in this article are solely those of the authors and do not necessarily represent those of their affiliated organizations, or those of the publisher, the editors and the reviewers. Any product that may be evaluated in this article, or claim that may be made by its manufacturer, is not guaranteed or endorsed by the publisher.
